# A novel machine learning model for perimeter intrusion detection using intrusion image dataset

**DOI:** 10.1371/journal.pone.0313890

**Published:** 2024-12-19

**Authors:** Shahneela Pitafi, Toni Anwar, I Dewa Made Widia, Zubair Sharif, Boonsit Yimwadsana

**Affiliations:** 1 Computer & Information Sciences Department (CISD), Universiti Teknologi PETRONAS, Bandar Seri Iskandar, Perak, Malaysia; 2 Faculty of Vocational Studies, Universitas Brawijaya, Malang, East Java, Indonesia; 3 Computer Science Academic Group, Faculty of Information and Communication Technology, Mahidol University, Salaya, Nakhon Pathom, Thailand; University of Lagos Faculty of Engineering, NIGERIA

## Abstract

Perimeter Intrusion Detection Systems (PIDS) are crucial for protecting any physical locations by detecting and responding to intrusions around its perimeter. Despite the availability of several PIDS, challenges remain in detection accuracy and precise activity classification. To address these challenges, a new machine learning model is developed. This model utilizes the pre-trained InceptionV3 for feature extraction on PID intrusion image dataset, followed by t-SNE for dimensionality reduction and subsequent clustering. When handling high-dimensional data, the existing Density-Based Spatial Clustering of Applications with Noise (DBSCAN) algorithm faces efficiency issues due to its complexity and varying densities. To overcome these limitations, this research enhances the traditional DBSCAN algorithm. In the enhanced DBSCAN, distances between minimal points are determined using an estimation for the epsilon values with the Manhattan distance formula. The effectiveness of the proposed model is evaluated by comparing it to state-of-the-art techniques found in the literature. The analysis reveals that the proposed model achieved a silhouette score of 0.86, while comparative techniques failed to produce similar results. This research contributes to societal security by improving location perimeter protection, and future researchers can utilize the developed model for human activity recognition from image datasets.

## 1 Introduction

**Fig 1:**
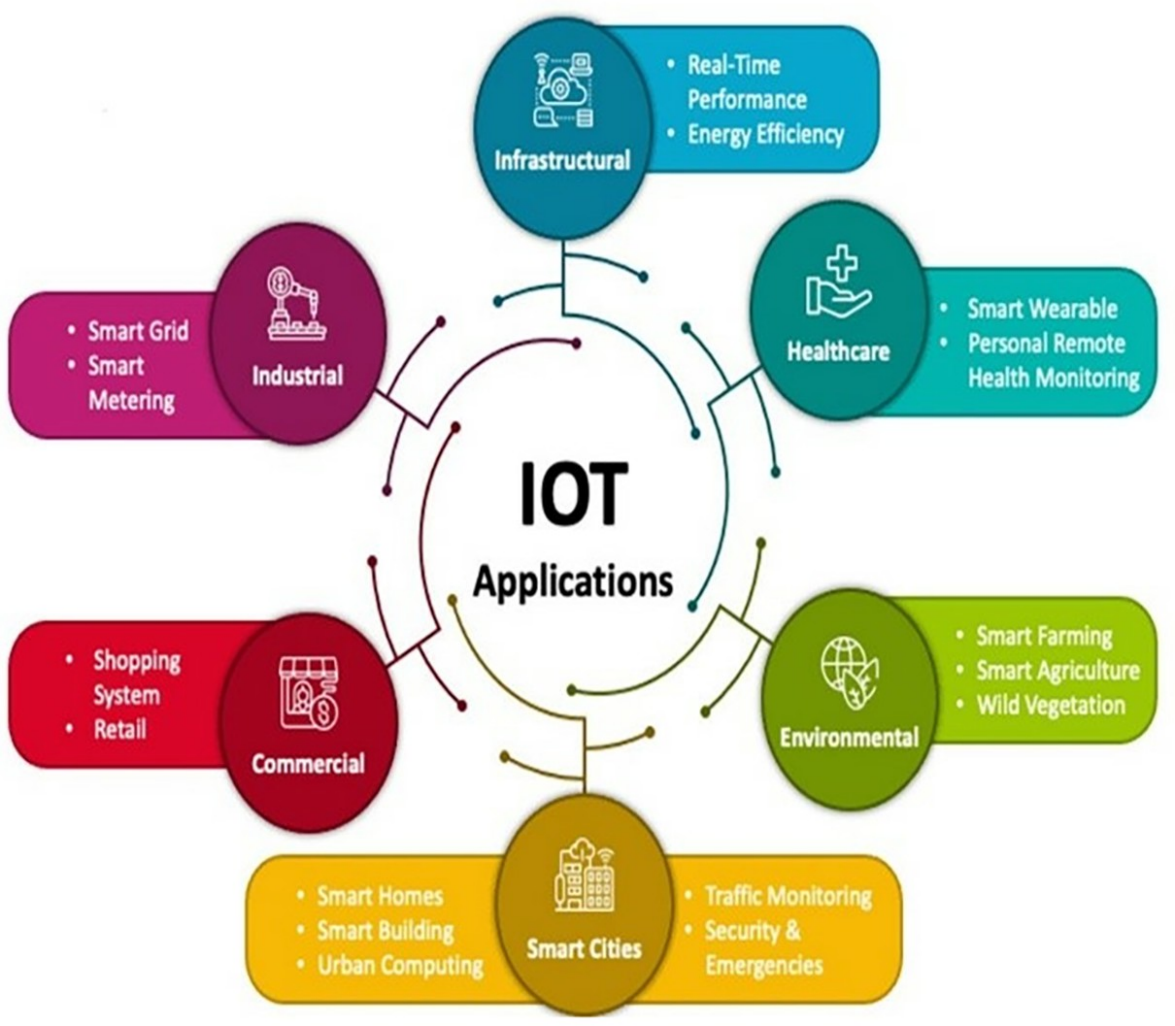
Illustrates the applications of IoT.

The Internet of Things (IoT) has infused everyday life, contributing to social sensing and information collection in a variety of fields such as smart transportation, intelligent cities, healthcare, and smart homes. Numerous smart-sensor nodes are installed inside specified domains to manage the sensing, processing, and transmission of diverse data kinds, including environmental characteristics, traffic density, human activity, and health data [1]. Fig 1 depicts a high-level overview of IoT applications. In addition, IoT is used in intrusion detection systems such as perimeter intrusion detection, network intrusion detection, and cloud intrusion detection [2].

PIDS are essential for protecting vital organizations by preventing illegal entry and hypothetical protection breaches. These methods are specifically engineered to identify and address intrusions occurring along the perimeter of a facility, acting as the initial line of defense against external threats. Nevertheless, with the evolving complexity and diversity of security threats, conventional PIDS approaches frequently encounter challenges, leading to issues such as false alarms, overlooked detections, and compromised security [3, 4]. The progress of vision-based technologies has seen substantial advancements throughout the previous two centuries [5].

PID is a task aimed at identifying the presence of an unauthorized object within a secure outdoor area during specific time intervals [6–8]. To maintain the security of an outdoor space, cameras continuously record footage in this environment. The distinction of being an outdoor setting is essential due to challenges such as changing weather conditions, variations in light, and the presence of insects and animals, unlike indoor environments [9, 10]. The user defines the protected zone at the location, potential intrusion elements, and the specific times when the system should offer protection, such as exclusively during nighttime.

The widespread deployment of cameras in critical settings such as banks, grocery stores, and prominent sidewalks has significantly contributed to the advancement and assessment of these systems. Among the noteworthy and relevant applications for intelligent vision systems is visual monitoring [11]. This encompasses various initiatives, including object identification, object monitoring, and the identification of unusual behaviors, all achievable through visual inspection of a scene [12].

The duty of intrusion detection is inextricably linked to other aspects of surveillance, and numerous modern solutions target one of these supplemental duties inside the monitoring system. However, only a few solutions address the topic of PIDS in depth [13, 14]. Given that neglecting intrusions is regarded as a serious failure for PIDS [14], current techniques prioritize boosting detection rates even at the risk of some false alarms.

To determine the success of the suggested technique, extensive testing, and comparative analysis were conducted. The conclusions of this investigation are presented, emphasizing the good productivity of the machine learning-driven technique the results of proposed technique and comparative techniques are evaluated by using Adjusted Rand Index (ARI), Fowlkes-Mallows Index (FMI) and silhouette score these are the evaluation metrics for the clustering approaches the details are presented in methodology section. After that k-NN classification algorithm is applied to clustered data and labeled images to classify the various intrusion activities. Finally, the approach’s capacity to identify and distinguish intrusions is demonstrated by evaluation measures such as classification accuracy, precision, recall, and F1 score. As a result, this adds to a betterment in major infrastructure security. Moreover, this study considers only six types of intrusion activities, with an additional class considered as non-intrusion, resulting in a total of seven classes.

The remainder of the document is structured as follows: A selection of the well-known and closely related work is covered in Section 2. In Section 3, the suggested novel PIDS Model is presented in all its depth and with all the essential explanations. In Section 4, the experimental results are discussed, and the offered work is verified. In section 5 we concluded our research in Section 5.

## 2 Related work

A study conducted in [15] exclusively delved into advanced signal processing, proposing an algorithm for event categorization. This algorithm analyzed both static and dynamic signals related to vibrations in a perimeter security system. Utilizing wavelet packet decomposition and constructing a multiclass classification tree with support vector machines, it achieved a commendable 94.6 percent recognition rate for vibration signals across nine distinct events.

In a related study [8], authors demonstrated the usage of autoencoders. However, no approach was provided for selecting the threshold. To ensure IoT network safety, Intrusion Detection Systems (IDSs) play a crucial role in identifying intruders. However, deploying complex IDSs in IoT devices is often impractical due to resource constraints. IDSs encompass signature-based and anomaly-based types, with the former recognizing threats based on predefined patterns, and the latter learning typical system behavior to detect anomalies.

Martin Ester and colleagues proposed Traditional Density-Based Spatial Clustering of Applications with Noise (T-DBSCAN) [22], dependent on neighborhood parameters. Recent enhancements focused on clustering analysis, as seen in [23], where a modified DBSCAN was developed. Additionally, authors in [24, 25] proposed a modified DBSCAN method for gene expression data, and researchers in [26] suggested an adaptive DBSCAN method for constellation reconstruction and modulation detection. A binary differential evolution method was proposed by [27] as a means of improving DBScan parameters (Eps) and figuring out the Eps interval. To tackle the problem of improving two crucial DBScan parameters, The Authors of [28] proposed a unique method based on combining Particle Swarm Optimization (PSO) with DBScan. A hierarchical clustering technique was presented by [18] to provide a range of values and improve the Eps parameter. To speed up the procedure overall, researchers in [29] developed a novel model for the automated selection of Eps and MinPts using the K-distance graph approach. In addition, The OPTICS approach was presented by [30] and is intended to locate clusters with different densities by keeping track of the reachability and core distances of every site. If a point has at least MinPts points in its Eps-neighborhood, it is said to be core; its core distance tells us how far away it is from the point that is closest to it. The bigger the distance between two locations or the core distance of point p yields the reachability distance. The research did find a drawback, though, in automatically and efficiently obtaining both intrinsic and conventional clustering information [30]. In order to overcome the difficulty of finding clusters in high-dimensional data [31], suggested a method that could handle a variety of sizes, shapes, and densities [31]. The method looks at each data point’s closest neighbors, creating clusters surrounding points based on common. The [31] demonstrated increased performance on benchmark datasets by introducing a revised version of DBSCAN, called EPDCA, to detect clusters with varied forms and sizes [31].

The approximation adaptive density-based spatial clustering of applications with noise (AA-DBSCAN) was proposed by [32], with an emphasis on minimizing computation for parameter estimation using e-distance for finding clusters. GCMDDBSCAN was developed by [33] to improve clustering performance on big datasets. A multi-agent system-based technique called FlockStream was introduced by [34]. It performed well on both illusionary and real datasets. The D-Stream model was established by [35] as a density-based method for grouping data in real-time. Labeled data or paired restrictions are used by semi-supervised clustering algorithms, such as Multi-density-based spatial clustering of applications with noise (MDBSCAN) by [36], density-based clustering with constraints (C-DBSCAN) by [37], and Semi-supervised density-based clustering (SSDBSCAN) by [38], to increase clustering efficiency. However, multi-density data and clusters with different pairwise restrictions provide difficulties for these techniques. A very well semi-supervised clustering variation of K-means, the MPCKmean method by [39] is restricted in its ability to handle clusters of varying densities and sizes, but it is successful for tackling massive datasets. In order to overcome the density variation problem with DBSCAN [40], developed the VDBSCAN algorithm which is intended for the analysis of datasets with different densities. The basic idea behind VDBSCAN is to use pre-processing techniques to get many values for the Eps parameter from a k-dist plot, and then use the standard DBSCAN algorithm. Through the use of several Eps values, the method finds multiple densities of clusters at the same time. The process involves five stages: (1) finding, storing, and splitting k-dist plots for each object; (2) counting the number of densities from the k-dist plot; (3) choosing the Eps parameters automatically for each density; (4) scanning the dataset and grouping different densities based on the matching Eps values; and (5) displaying the valid clusters. Further, the Authors of [41] presented the foundation of LDBSCAN as the local-density-centered notion of clusters. This approach gives it an edge over previous density-based clustering algorithms since it streamlines the factor review process and uses LOF for vibration identification. In addition, a novel clustering approach was presented that overcomes the restrictions of DBSCAN and achieves a significant runtime acceleration in comparison to prior improvements. The user guide’s assessment technique has limits when it comes to the i-LIDS dataset; alerts that are more than 10 seconds old are penalized without taking the duration of the incursion into account [42].

This thorough review emphasizes the many strategies and difficulties associated with intrusion detection and clustering methods, highlighting the dynamic security environment on the Internet of Things and its associated applications further comparison is presented in Table 1.

**Table 1:** Presents the comparison of various density based algorithms.

S#	Technique	Noise Handling	Parameter setting	Complexity	Sensitivity	Performance	Cluster quality
1	K-DBSCAN [16]	Noise at the borders of merged clusters can affect the final outcome.	It needs four parameters, MinPts, Eps mixed with K-means so need its cluster number and K-means iteration	Increased complexity in understanding and deploying the algorithm.	—	Might require high memory for extremely large datasets.	Maintains clustering quality close to that of traditional DBSCAN with reduced parameter usage.
2	MDBSCAN [17]	—	May require careful tuning of parameters k and t for optimal performance.	Potentially higher complexity with larger datasets due to multi-step process.	—	Performance may degrade with improperly set parameters or very large datasets.	Requires more computation of all resources due to multiple processing steps.
3	DBHC [18]	—	Complexity in understanding and implementing the method due to automatic parameter setting.	Increased computational complexity, particularly for large datasets due to multiple runs of DBSCAN.	Requires careful tuning of the number of clusters.	Performance can degrade if the assumption about distribution is not met.	Might generate large number of clusters or merged, complicated shapes depending on the scenario.
4	ADBSCAN [19]	The adaptive approach may initially misclassify dense clusters as noise, requiring further iterations to correct.	Still requires careful selection of MinPts to ensure effective clustering.	Increased complexity in understanding how adaptive Eps calculations affect clustering outcomes.	The iterative process for adjusting Eps may not scale efficiently with extremely large datasets.	Consumes more time than traditional DBSCAN due to iterative adjustments of Eps and cluster merging.	Requires additional computational steps to merge clusters, which can complicate the clustering process.
5	VDCA [20]	Might mistakenly classify sparse clusters as noise.	The choice of k and SR is crucial and can be sensitive to dataset specifics.	Complex parameter tuning required to handle varied data densities.	Potential increase in computational load due to the calculation of distances for multiple neighbours.	Requires careful implementation to ensure that the dynamic density calculation is correctly managed.	Risk of misclassifying boundary objects if similarity thresholds are not well-defined.
6	Fast-DBSCAN [21]	Potential for misclassifying sparse clusters as noise in some contexts.	Initial parameter setting for DBSCAN still influences the outcome.	The method’s efficiency is sensitive to the distribution and density of data.	—	Requires additional implementation complexity to manage groups.	Requires two scans of the dataset, potentially increasing preprocessing time.

## 3 Proposed methodology

**Fig 2:**
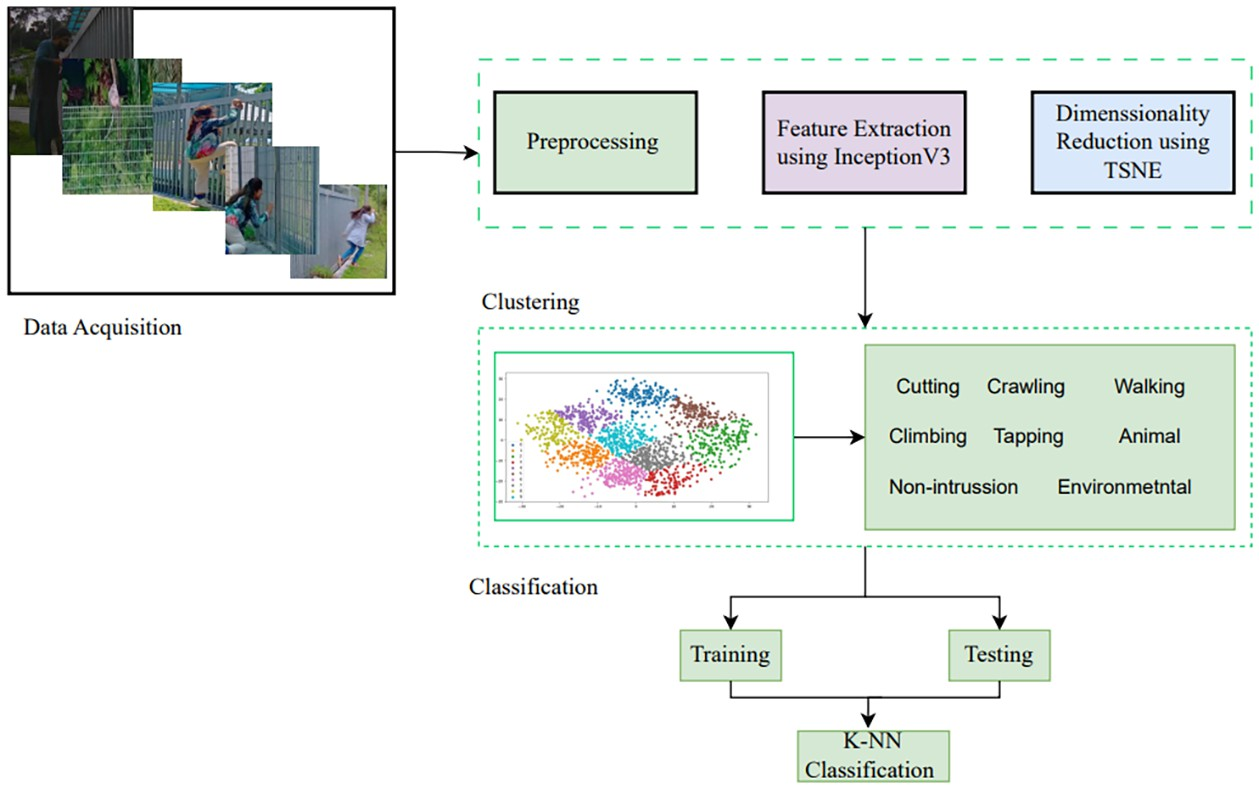
Proposed PIDS model.

This section presents the methodology of a novel PIDS model for perimeter intrusion detection systems. Where an enhanced DBSCAN algorithm is used, with our proposed method the existing issues in PID can be resolved and the accuracy of detection is increased along with the classification and identification of various intrusion activities. In this section, we have presented the steps of our proposed model named novel PID model using image dataset as illustrated in Fig 2. The PID image-based dataset for the evaluation of proposed technique is used. Further the methodology of this study is divided into two phases, one is clustering part, and the other is classification part. The outcome of phase one is the input for the second phase of the research details of methodology are given in the next section.

### 3.1 Pre-processing

Pre-processing involves removing duplicate images during this phase, a meticulous step was taken to enhance the quality of the dataset. Duplicate images were systematically identified and subsequently removed using their unique hash values. This crucial procedure ensured that the dataset maintained a high level of integrity and eliminated redundancy. By filtering out duplicate instances based on their hash signatures, we aimed to enhance the overall robustness and efficiency of the dataset, laying the groundwork for more accurate and reliable analyses in subsequent stages of our research.

**Fig 3:**
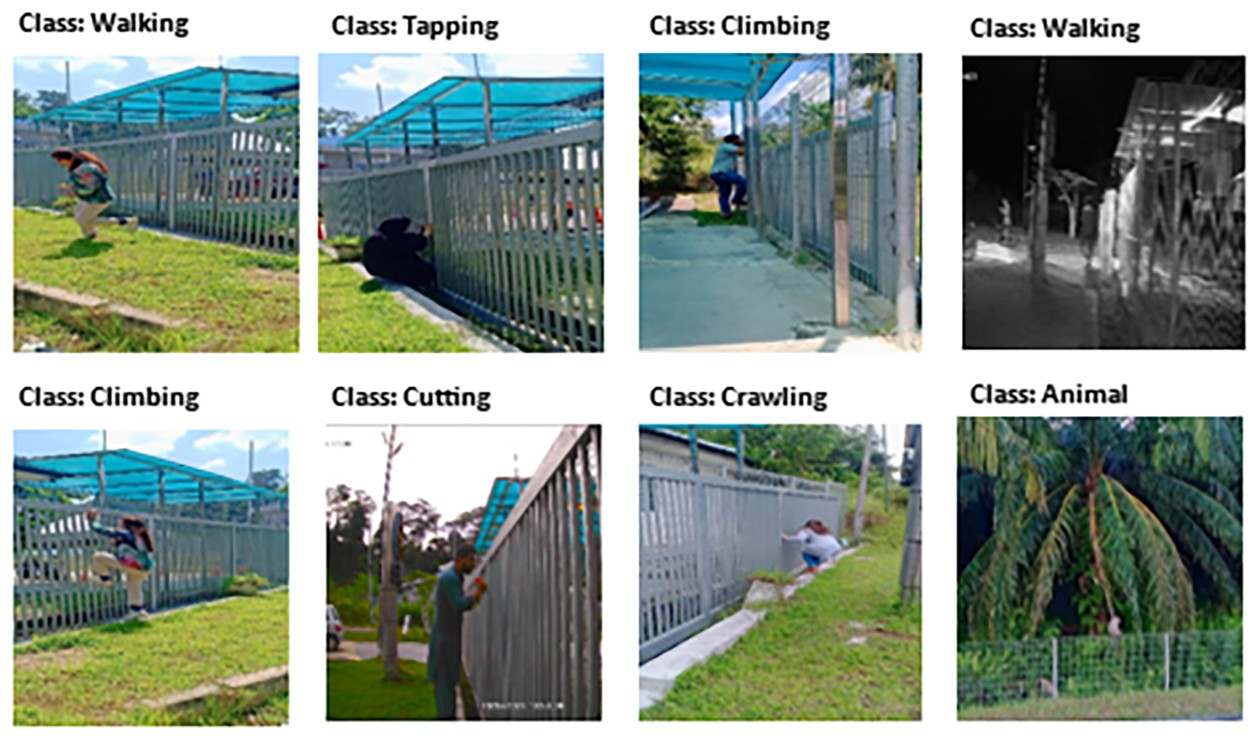
PID image dataset sample.

Data augmentation is a technique to increase the quantity and quality of dataset [43]. Additionally, to improve the generalization ability of the deep learning-based image classification model, data augmentation can be applied to both the training and validation sets [44]. A similar approach is followed in this study, and data augmentation has been applied to the whole dataset. The sample data is presented in Fig 3.

Data augmentation methods, such as geometric transformation, kernel filters, mixing images, random erasing, and transformation [43] are evaluated for the suitability of intrusion scene augmentation. Details are provided in Table 2.

**Table 2:** List of augmentations selected.

S#	Augmentation type	Post augmentation observation	Selection status
1	Vertical flip	Visual flip the image vertically	Selected
2	Horizontal flip	Visual flip the image horizontally	Selected
3	Brightness and contrast	Introducing a wide range of illumination	Selected
4	Random cropping	This may result in unnecessary objects in image	Selected
5	Rotation	Imitates optical sensors roll effect	Selected
6	Noise injection	Improves models generalization ability	Selected
7	Motion blur	Imitates optical sensors movements	selected
8	Sharpening	More appropriate for object detection	Selected

After the augmentation techniques applied the images increased to 7,962 total which comprises the seven classes as discussed in Table 3.

**Table 3:** Details of PID image dataset.

Class labels	Name of classes	Total Numbers of Extracted Images	Overall Percent Share
1	Climbing	1,197	15.03%
2	Crawling	1,114	13.99%
3	Tapping	1,068	13.41%
4	Cutting	1,202	15.09%
5	Walking	1,263	15.86%
6	Animal	1,080	13.56%
7	Non-intrusion	1,038	13.03%
	**Total**	**7,962**	**100%**

### 3.2 Feature extraction of images

After data augmentation, the feature extraction phase was executed using the pre-trained Google InceptionV3 model. This transfer learning approach allowed us to leverage the well-established features learned by InceptionV3 on a large dataset. By extracting these high-level features from augmented images, we aimed to capture intricate patterns and representations essential for effective intrusion detection. The utilization of pre-trained models like InceptionV3 enhances the efficiency of our model training process, empowering it with a rich set of discriminative features for improved performance in the classification task.

In Inception network such as InceptionV3, features for t-SNE (t-distributed Stochastic Neighbor Embedding) are extracted from the penultimate layer it is the layer just before the final classification layer. This layer provides high-level features that have been processed by the network but are not yet reduced to the final class probabilities. The penultimate layer contains a rich representation of the image, capturing high-level abstract features. These features are useful for tasks like clustering or dimensionality reduction, as they represent the most informative parts of the input data.

CNNs utilize a series of convolutional layers that apply convolution operations to the input images. The convolution operation involves sliding a small filter or kernel across the image, computing the dot product between the filter and the local receptive field of the image. This process helps in extracting local patterns and features. The resulting feature maps are then passed through non-linear activation functions such as ReLU (Rectified Linear Unit) to introduce non-linearity. Mathematically, the convolution operation can be expressed in Eq (1)

$$\begin{array}{c} \text{Feature}\;\text{Map}\left(i,j\right)=\sigma (\sum_{m}\sum_{n}\text{image}\left(i+m,j+n\right)\times \text{filter}\left(m,n\right)+b) \end{array}$$ (1)

Here, i and j denote the spatial coordinates of the feature map, *σ* is the activation function, image (i+m,j+n) represents the pixel values in the local receptive field of the image, Filter (m,n) represents the values of the convolution filter, and b represents the bias term.

InceptionV3 stands out for its efficiency, multi-scale feature extraction, and strong performance on large image datasets. Its ability to balance high accuracy with lower computational requirements makes it a solid choice for a wide range of image classification tasks instead of selecting other CNN models.

### 3.3 Dimensionality reduction with t-SNE for clustering

Following feature extraction with the InceptionV3 model, dimensionality reduction using t-SNE was executed for clustering. t-SNE effectively condensed the high-dimensional feature space, preserving data structure for insightful clustering analysis. This approach enhances interpretability, offering valuable insights into inherent patterns within the dataset.

The function for t-SNE can be mathematically represented in Eq (2) [45].

$$\begin{array}{c} c=\sum_{i}KL\left(P_{i}\|Q_{i}\right)=\sum_{i}\sum_{j}p_{ij}\;\text{log}\;\frac{p_{ij}}{q_{ij}} \end{array}$$ (2)

where:

- *p*_*ij*_ represents the similarity between data point *i* and data point *j* in the high-dimensional space,
- *q*_*ij*_ represents the similarity between the corresponding data points in the low-dimensional space,
- *P*_*i*_ and *Q*_*i*_ are the probability distributions in the high-dimensional and low-dimensional spaces, respectively.

The t-SNE algorithm iteratively minimizes this cost function to find an optimal low-dimensional representation of the data that preserves local structure and relationships from the high-dimensional space.

### 3.4 Proposed enhanced DBSCAN

**Fig 4:**
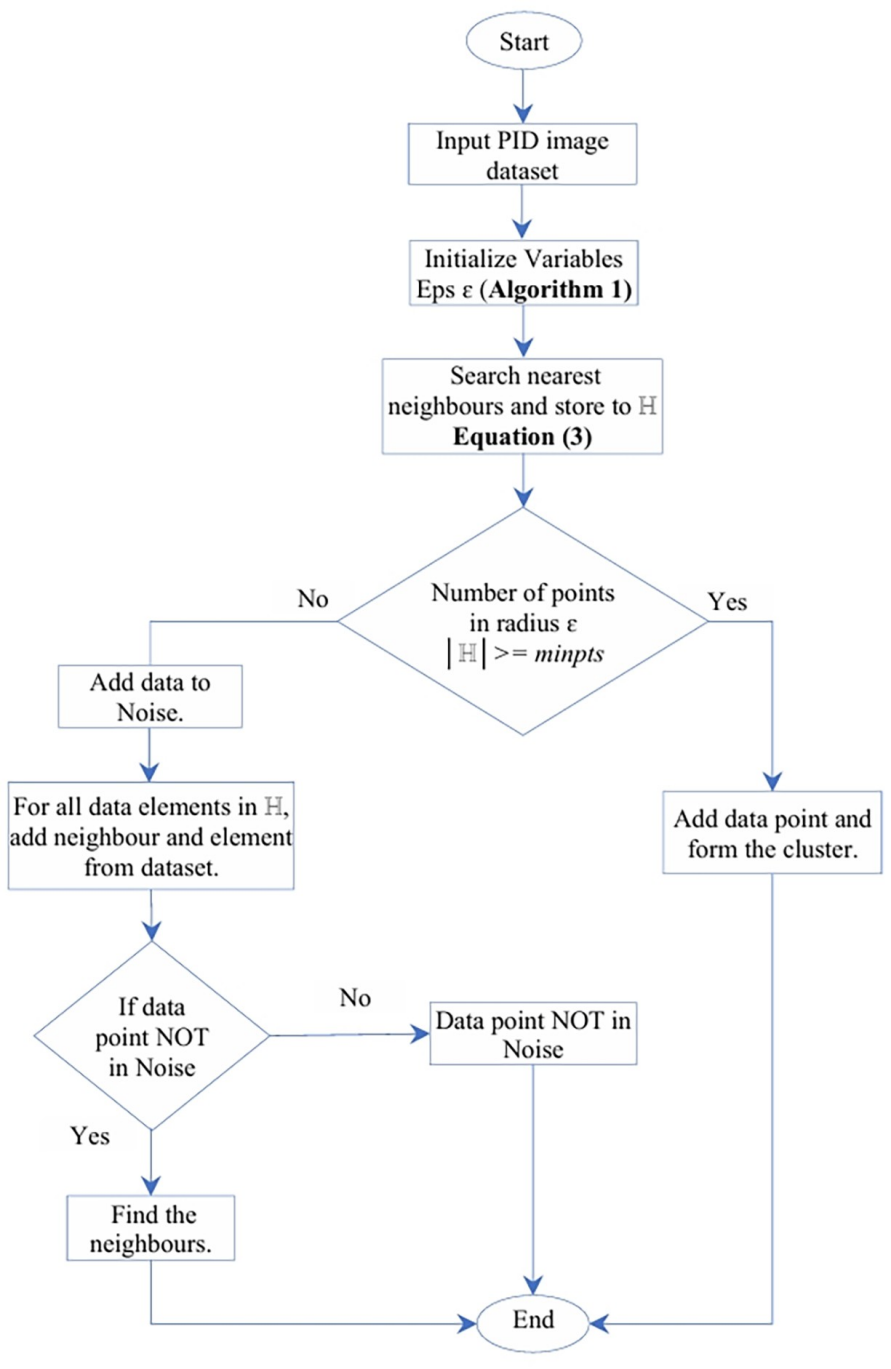
Proposed enhanced DBSCAN flow chart.

DBSCAN is a clustering algorithm reliant on the proximity of data points in the feature space, it groups data points based on their density, effectively discerning dense and less crowded regions to capture the inherent data structure. After extracting and reducing features, they are considered as data points, and DBSCAN computes their density, assigning clusters using user-defined thresholds for minimum density and distance parameters. In the traditional DBSCAN method users regulate values of epsilon and Nmin. To automate and eliminate human intervention, we introduce an estimation for parameter epsilon in Algorithm 1, utilized in our proposed enhanced DBSCAN method as depicted in Fig 4. This clustering procedure aids in differentiating real intrusions from non-intrusive scenarios, enabling researchers to identify meaningful clusters representing distinct patterns in the multi-dimensional data. The method significantly contributes to improving intrusion detection system accuracy by effectively distinguishing between various intrusion types and non-intrusive scenarios, as outlined in the results section.

$$\begin{array}{c} \|X_{a}-X_{b}{\|}_{M}=|x_{a1}-x_{b1}|+|x_{a2}-x_{b2}| \end{array}$$ (3)

**Algorithm 1** Epsilon parameter estimation *ε*

1: **Input:** dataset *A* = {*a*_1_, *a*_2_, …, *a*_*n*_}

2: **Variables:** *MinPts*   ⊳ *MinPts* are calculated by Eq (3)

3: **Calculate** distance between data points

4: **for** *P* in range [2, *n*] **do**   ⊳ data items

5:  Calculate *d*_*p*_ using Eq (3)   ⊳ For each detected object/feature point *P* in the image,

6:  Append (*P*, *d*_*p*_) to *d*_*p*_ values

7:  Polynomial fitting for *discrete function* *d*_*p*_   ⊳ To obtain continuous function coefficients

8:  **if** *S*^2^ < 0.99 **then**   ⊳ If *S*^2^ (fitting accuracy) is less than 0.99

9:   *P* = *P* + 1 and go to line 3   ⊳ Increase *P* and repeat from the polynomial fitting step

10:  **else**

11:   Adding correction   ⊳ Compute the correction factor based on the maximum value of *D*_*P*_

12:  **end if**

13:  Calculate the derivative   ⊳ Derive the first derivative of the corrected *D*_*P*_

14:  **if** *p*_0_ > *P* **then**   ⊳ Solve for *p*_0_ if *p*_0_ > *P*

15:   *P* = *P* + 1 and go to line 3   ⊳ Increase *P* and repeat

16:  **else**

17:   Calculate the estimated radius *ε*   ⊳ Calculated the estimated radius

18:  **end if**

19: **end for**

The Manhattan distance, also known as “city block distance,” calculates total distance measure as the sum of ranges from all characteristics for two variables Xa and Xb in d-space measurements. The Manhattan distance between the locations is defined as above.

### 3.5 Preparing the data for classification with K-NN

**Fig 5:**
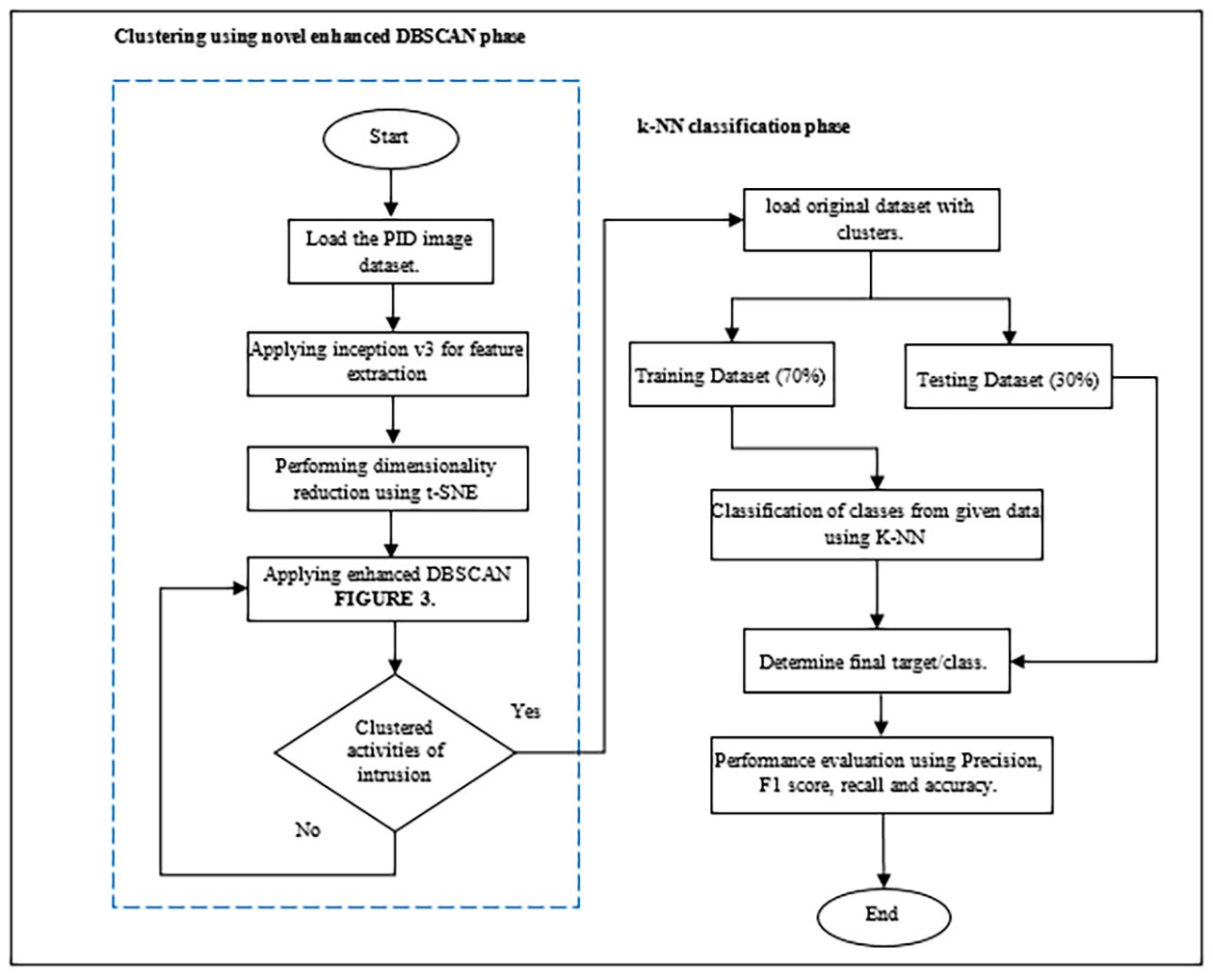
Flow chart of Novel enhanced DBSCAN based model.

Fig 5 presents the whole flow chart of the proposed model and Algorithm 2 introduces the proposed DBSCAN based model for PIDS, enhancing perimeter intrusion detection through advanced data processing and machine learning techniques. It starts by loading the PID image dataset, specialized for perimeter intrusion scenarios. Feature extraction is performed using the Inception v3 model, translating raw data into a structured feature space. To manage complexity, t-SNE is applied for dimensionality reduction. The algorithm then uses an enhanced DBSCAN, (Algorithm 1), for density-based spatial clustering, which effectively identifies unusual activities or security breaches without needing a predefined number of clusters. Clustering results are evaluated by using the evaluation metrics ARI, FMI and silhouette score as detailed in Eqs (4), (5), (6) and (7). After clustering, the algorithm proceeds to classification using the k-Nearest Neighbors (k-NN) approach. The cluster outcomes are treated as labels for training the k-NN model. The dataset is split into 70% for training and 30% for testing.

#### Adjusted Rand Index (ARI)

ARI measures the similarity between two clustering’s by adjusting for the chance grouping of elements. It considers all pairs of samples and checks if they are in the same or in the different clusters in predicted and true clusters as mathematically presented in Eqs (4) and (5).

$$\begin{array}{c} ARI=\frac{Ri-\text{Expected}\;Ri}{\text{max}(Ri)-\text{Expected}\;Ri} \end{array}$$ (4)

where Ri (Rand Index) is given by:

Ri=a+b(n2) (5)

**Algorithm 2** Novel enhanced DBSCAN based model for PIDS

1: **Input:** load the PID image dataset

2: Applying inception v3 for feature extraction

3: Performing dimensionality reduction using t-SNE

4: **Applying enhanced DBSCAN** see Algorithm 1

5: Outcome of enhanced DBSCAN

6: **K-NN classification phase**

7: **Input:** load original dataset with clusters

8: *X* = *data*.*data*

9: *Y* = *data*.*target*

10: **Split the dataset into training and testing sets.**

11: • 70% of data goes to training set

12: • 30% of data goes to testing set

13: **for** each instance in the test data **do**

14:  Calculate the distance from each instance in the train data

15:  **for** i, *train*_*instance* in enumerate(*X*_*train*) **do**

16:   *dist* = *Euclidean*(*test*_*instance*, *train*_*instance*)

17:   distances.append((dist, i))

18:   distances.sort(key = lambda x: x[0])   ⊳ Sort distances and get the indices of the k-NN

19:   **Find the majority class among the data elements.**

20:   Return the predicted class.

21:  **end for**

22: **end for**

23: Measure the performance by using validation metrics from equation

24: **End**

And *a* is the number of pairs of elements that are in the same cluster in both the predicted and true clusters, *b* is the number of pairs in different clusters in both, and (n2) is the total number of pairs.

#### Fowlkes-Mallows Index (FMI)

FMI is a measure of the similarity between the true cluster assignments and the predicted cluster. It is particularly useful for evaluating the precision and recall of clustering results, which are critical aspects of clustering algorithms.

$$\begin{array}{c} FMI=\sqrt{\frac{TP}{TP+FP}\cdot \frac{TP}{TP+FN}} \end{array}$$ (6)

where TP (True Positives) is the number of pairs correctly clustered together, FP (False Positives) is the number of pairs incorrectly clustered together, and FN (False Negatives) is the number of pairs incorrectly not clustered together.

#### Silhouette score

The Silhouette Score evaluates how similar a sample is to its own cluster compared to other clusters. Silhouette Score is a metric used to evaluate the quality of a clustering solution. It measures how similar each point in one cluster is to points in the same cluster compared to points in other clusters. The Silhouette Score provides insight into how well the clusters are separated and how compact they are.

$$\begin{array}{c} s\left(i\right)=\frac{b(i)-a(i)}{\text{max}(a(i),b(i))} \end{array}$$ (7)

where *a*(*i*) is the average distance from the *i*-th sample to all other points in the same cluster, and *b*(*i*) is the minimum average distance from the *i*-th sample to points in a different cluster. The Silhouette Score is the mean *s*(*i*) for all samples.

The k-NN classifier predicts the class of test instances by calculating Euclidean distances and identifying the nearest neighbors. The classification categorizes data into intrusion activities, validating the proposed algorithm.

As presented in Table 4 the proposed enhanced DBSCAN is performing better due to its novel enhancement which has not been done in other variants of DBSCAN. Furthermore, the study’s findings indicate that the proposed technique significantly improves the detection and classification of intrusion activities. This is particularly evident in the comparative performance metrics, where the proposed model consistently outperformed other comparative models.

**Table 4:** Overall comparison of proposed enhanced DBSCAN with other variants of DBSCAN.

S#	Algorithm	Noise Handling	Parameter setting	Cluster quality	Limitations/ Performance
1	K-DBSCAN [16]	Noise at the borders of merged clusters are affecting the final outcome.	It needs four parameters, *MinPts*, Eps mixed with K-means so need its cluster number and number of K-means iteration.	Maintains clustering quality close to that of DBSCAN with reduced computational resources.	Requires high memory for large datasets.
2	MDBSCAN [17]	—	Require careful tuning of parameters k and t for optimal performance.	Requires more computational resources due to multiple processing steps.	Performance degrades due to improperly set parameters and multi-step process.
3	DBHC [18]	—	Complexity in understanding and implementing the method due to automatic parameter setting.	Might generate a large number of clusters that need to be merged, complicating the process.	Requires careful tuning of the number of clusters. Performance degrades due to multiple runs of DBSCAN.
4	ADBSCAN [19]	Initially misclassifies dense clusters as noise, requiring further iterations to correct.	Still requires careful selection of *MinPts* to ensure effective clustering.	Requires additional computational steps to merge clusters, which can complicate the clustering process.	The iterative process for adjusting Eps does not scale efficiently with larger datasets.
5	Enhanced DBSCAN (Proposed)	Handles noise well and detect it.	Epsilon is calculated by parameter estimation method and *MinPts* are dynamically calculated by the Manhatton distance formula	Generated clusters as required total seven cluster.	It generated required clusters and performed better in terms of silhouette score evaluation metrics.

## 4 Experimental results

**Fig 6:**
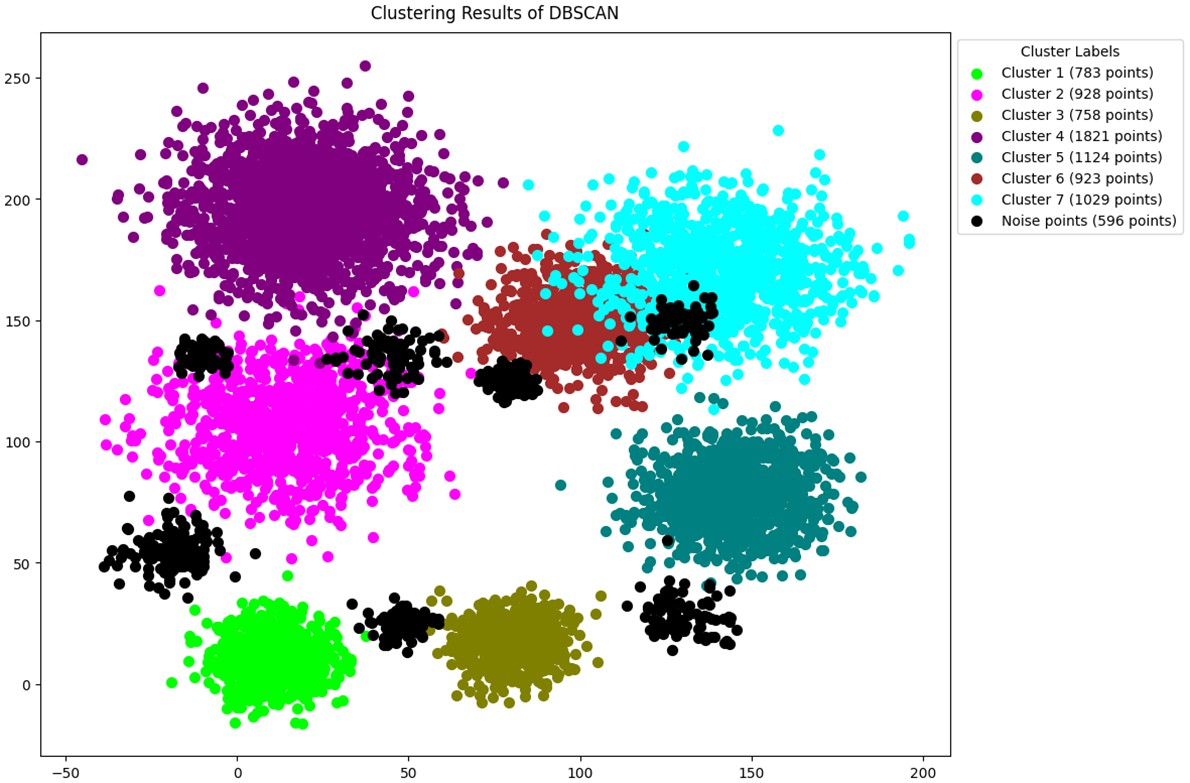
Results of DBSCAN clustering.

**Fig 7:**
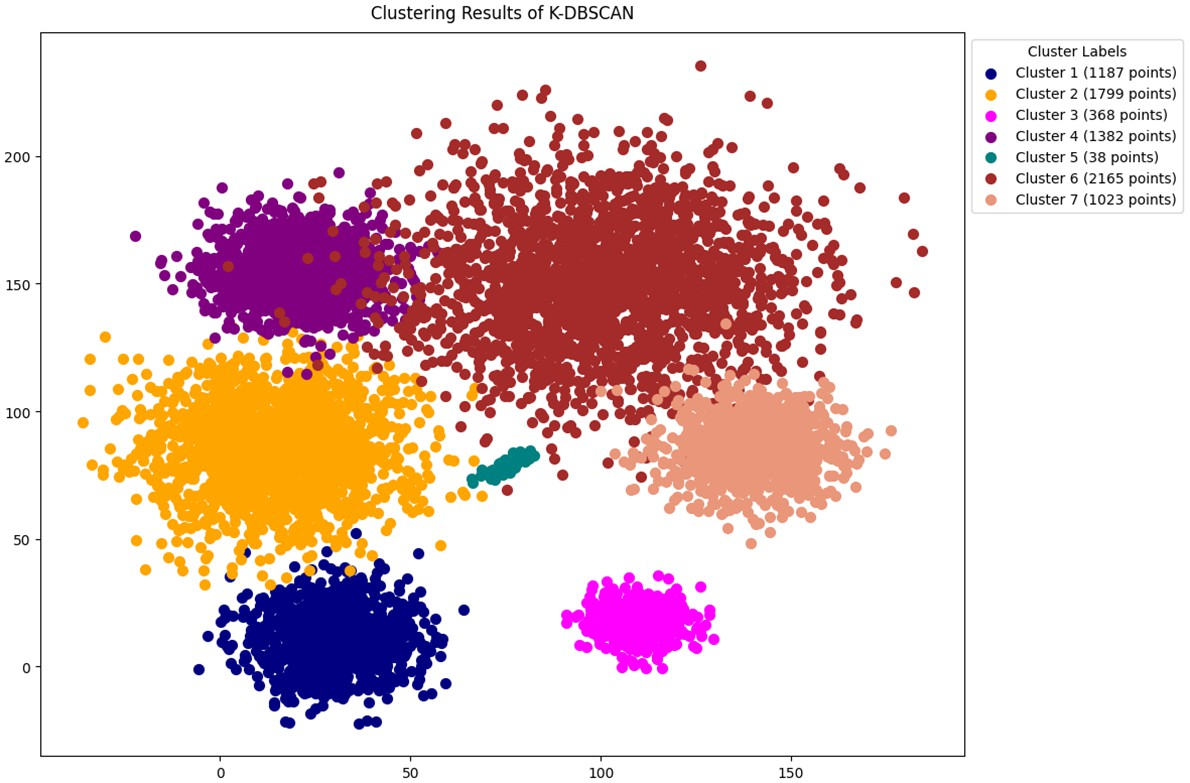
Results of K-DBSCAN clustering.

**Fig 8:**
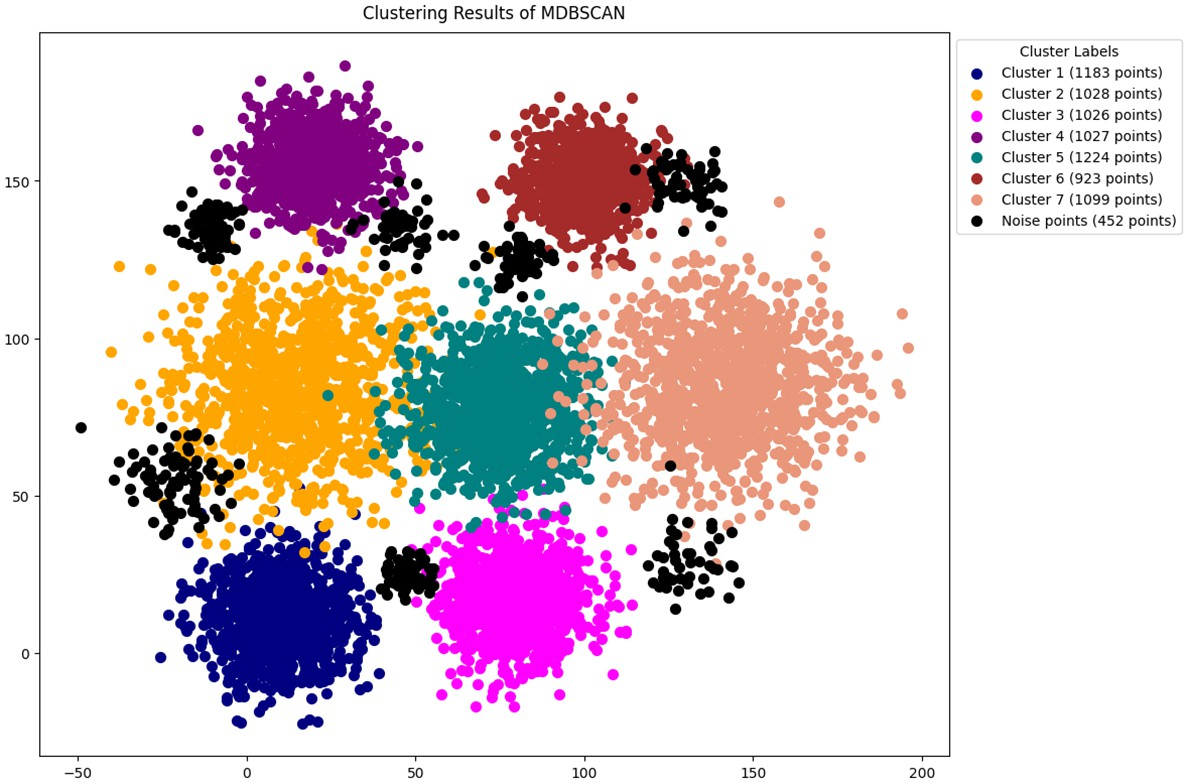
Results of MDBSCAN clustering.

**Fig 9:**
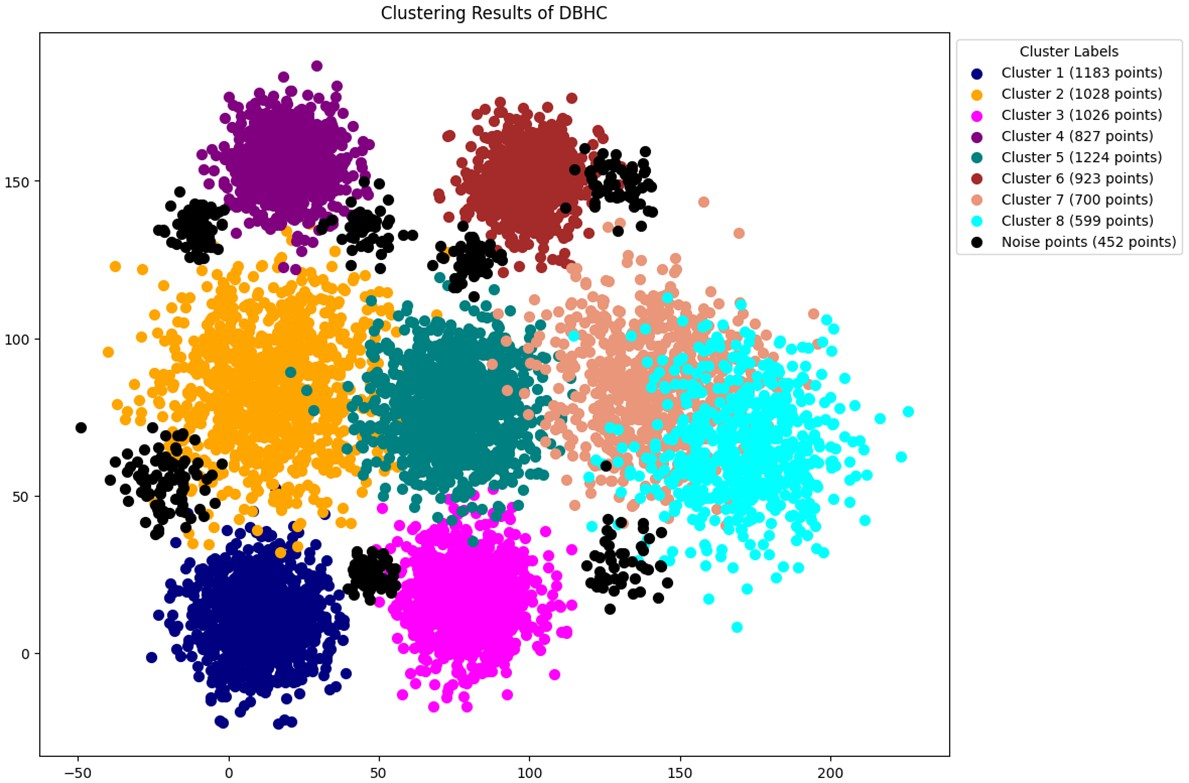
Results of DBHC clustering.

**Fig 10:**
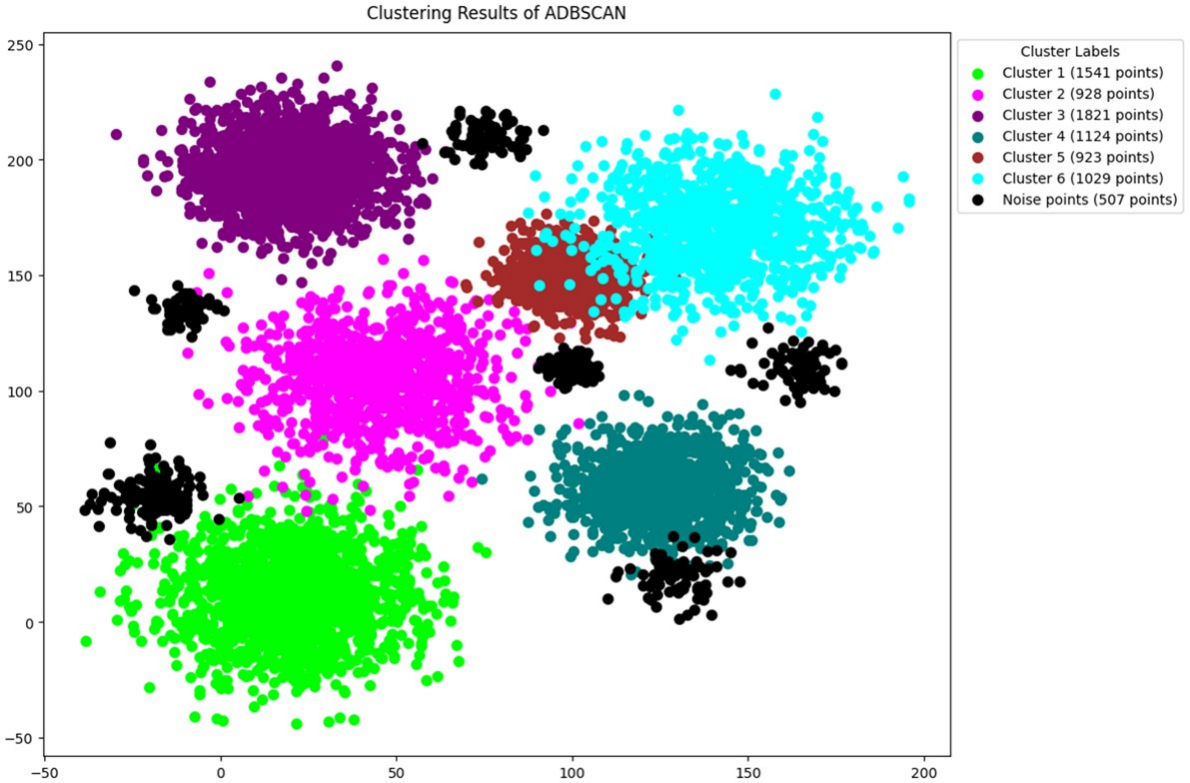
Results of ADBSCAN clustering.

In this study, the methodology presented in section 3 is followed, The features are selected by using python [46]. The original DBSCAN, K-DBSCAN, MDBSCAN, DBHC and ADBSCAN along with our proposed novel enhanced DBSCAN algorithm is applied to PID image dataset results of clustering visualization are presented in Figs 6–10 respectively.

Fig 6 illustrates the clustering results of DBSCAN, which segmented into seven distinct clusters, highlighting Cluster 4, the largest, contains 1,821 points, while Cluster 5 includes 1,124 points, similarly cluster 7 contains 1029 points Conversely, smaller clusters like Cluster 1 and Cluster 3, with only 783 and 758 points respectively. The large clusters may contain datapoints of other intrusion activities therefore the silhouette score is compromised. Furthermore, DBSCAN detected 596 noise data points, it may misclustered intrusion activities as noise therefore the clustering results are compromised.

Fig 7 illustrates the clustering results of K-DBSCAN, it made seven distinct clusters, presenting cluster 6, the largest, contains 2,165 points, while cluster 2 includes 1,799 points, Conversely, smaller clusters like cluster 3 and cluster 5, with only 368 and 38 points respectively. Furthermore, K-DBSCAN ignores noise data, therefore it does not detect noise values, which include the outlier of the dataset in clusters, therefore the clustering results are compromised.

The MDBSCAN clustering graph, shown in Fig 8, illustrates a dataset segmented into multiple clusters and noise, demonstrating diverse groupings of data points. Notably, cluster 1, contains 1183 points, cluster 6 has less datapoints unlike the ground truth datapoints. MDBSCAN contains 452 noise points, highlights variability and outliers.

Fig 9 presents the visualization of DBHC clustering applied to a PID image dataset reveals a diverse distribution of data points across several clusters, with sizes ranging from 599 to 1,224 points, and identifies 452 points as noise.

The ADBSCAN clustering output is depicted in Fig 10, where graph reveals six clusters only. In contrast, clusters like Cluster 2 and Cluster 5, holding only 928 and 923 points respectively.

Evaluation metrics include Precision, recall, F1 score and accuracy. Where Precision is the ratio of true positive predictions to the total predicted positives. It measures the accuracy of the positive predictions as represented in (8). Similarly, Recall is the ratio of true positive predictions to the total actual positives. It measures the model’s ability to capture all the positive instances given in (9), moreover, The F1 score is the harmonic means of precision and recall. It provides a balance between precision and recall, especially when there is an imbalance between classes see (10), lastly Accuracy is the ratio of correctly predicted instances to the total instances. It provides an overall measure of model performance presented (11).

$$\begin{array}{c} \text{Precision}=\frac{TP}{TP+FP} \end{array}$$ (8)

$$\begin{array}{c} \text{Recall}=\frac{TP}{TP+FN} \end{array}$$ (9)

$$\begin{array}{c} F_{1}\;\text{Score}=2*\frac{\text{Precision}*\text{Recall}}{\text{Precision}+\text{Recall}} \end{array}$$ (10)

$$\begin{array}{c} \text{Accuracy}=\frac{TP+TN}{\text{total}\;\text{instances}} \end{array}$$ (11)

In these equations:

True Positives (TP): Instances correctly predicted as positive.

False Positives (FP): Instances incorrectly predicted as positive.

False Negatives (FN): Instances incorrectly predicted as negative.

True Negatives (TN): Instances correctly predicted as negative.

**Fig 11:**
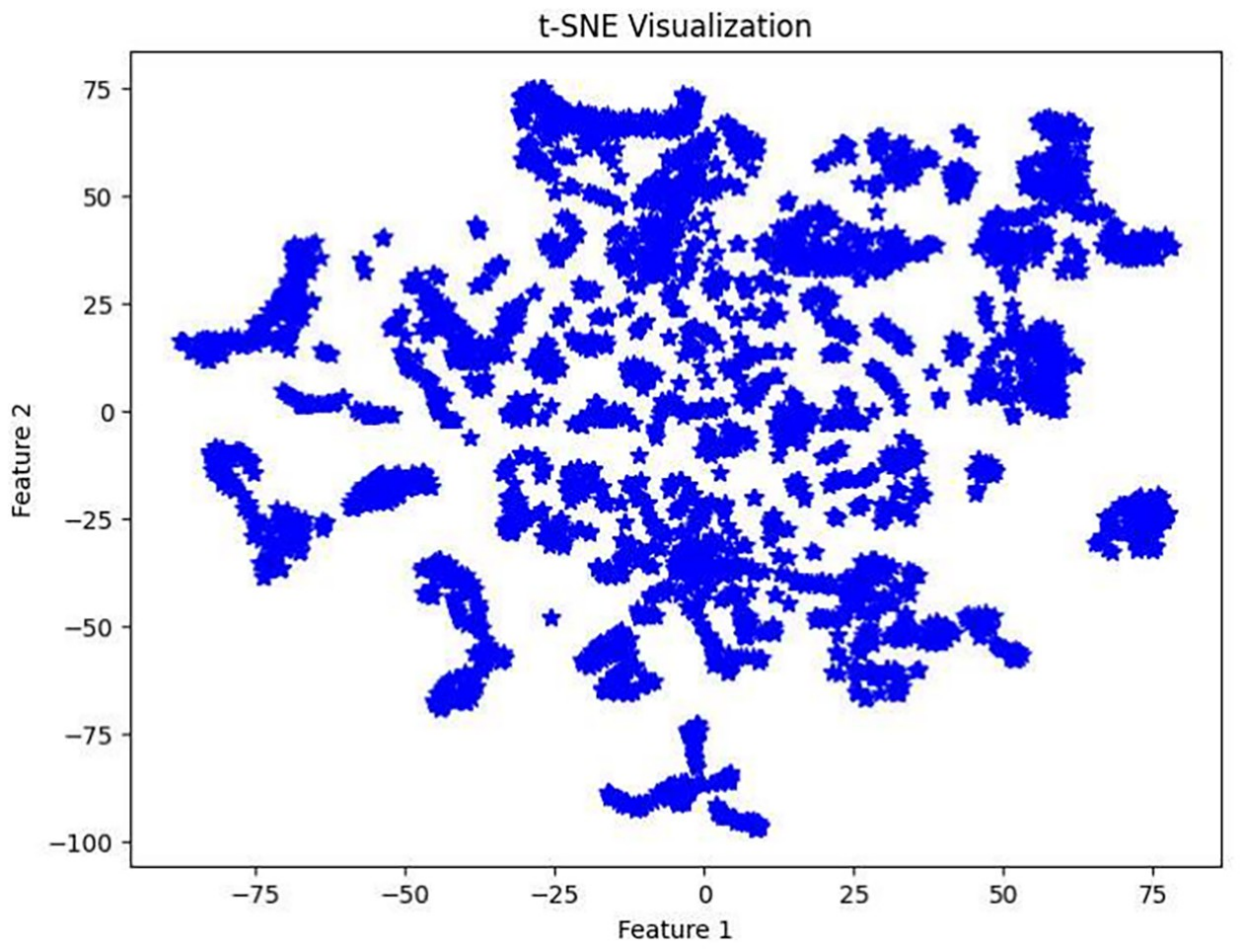
Visualization of t-SNE.

**Fig 12:**
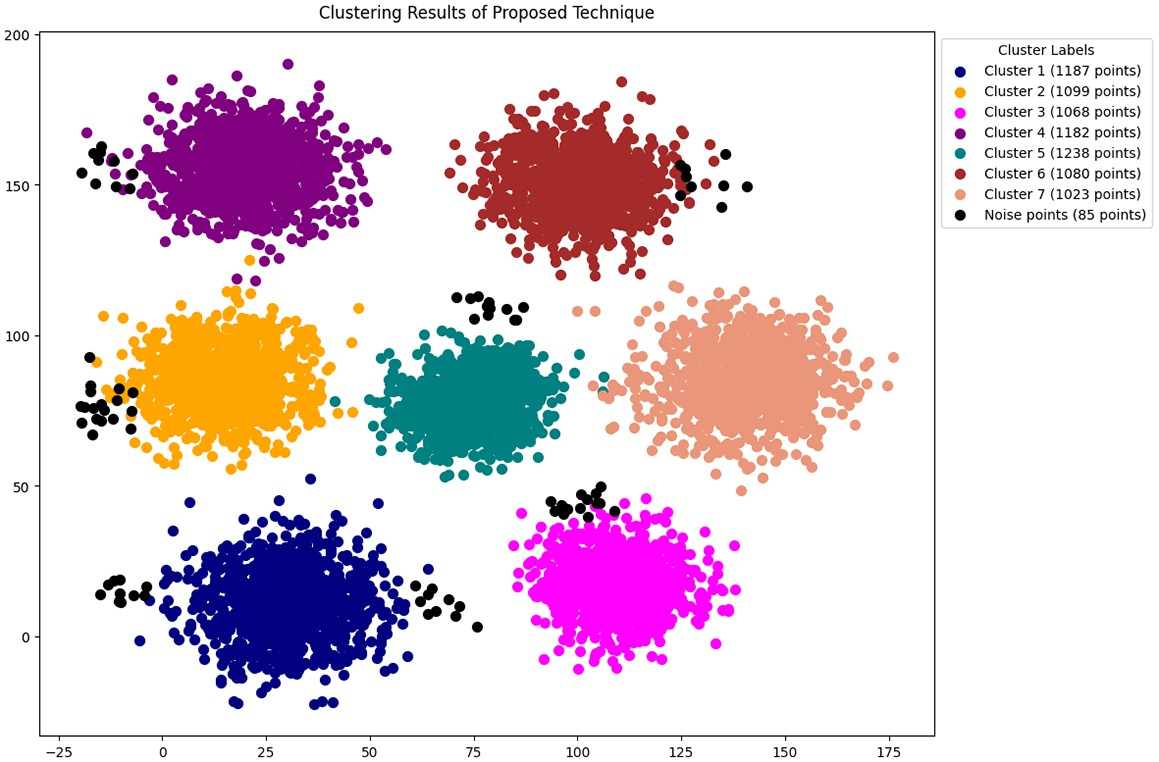
Results of proposed enhanced DBSCAN clustering.

Results obtained from dimensionality reduction with t-SNE are shown in Fig 11. The results from the proposed technique clustering algorithm is presented in Fig 12 applied to a dataset of 7,962 images across seven classes as required. It shows several clusters (labeled from cluster 1 to cluster 7), each in different colors, indicating groups of images similar based on the features analyzed. cluster 1 comprises 1,187 points, and the sizes of other clusters vary, suggesting varying degrees of similarity within the dataset. Additionally, the proposed algorithm has identified 85 points as noise these are outliers that do not fit well into any cluster due to their distinct features. This analysis shows points presented in graphs represent the number of images here each point represents an image. including both the identified clusters and noise, as shown in Fig 12 the clusters are well separated and there is no overlapping. As presented in Table 3, we have a total of seven categories of intrusions. Therefore, the seven different clusters correspond to the seven types of intrusions, as shown in the ground truth values.

**Fig 13:**
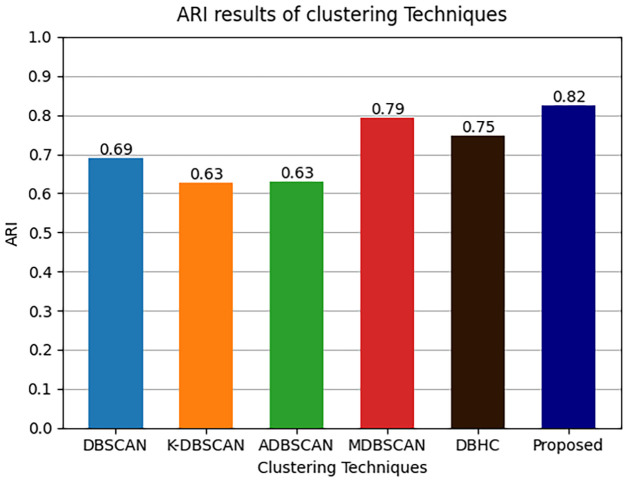
ARI metrics results.

**Fig 14:**
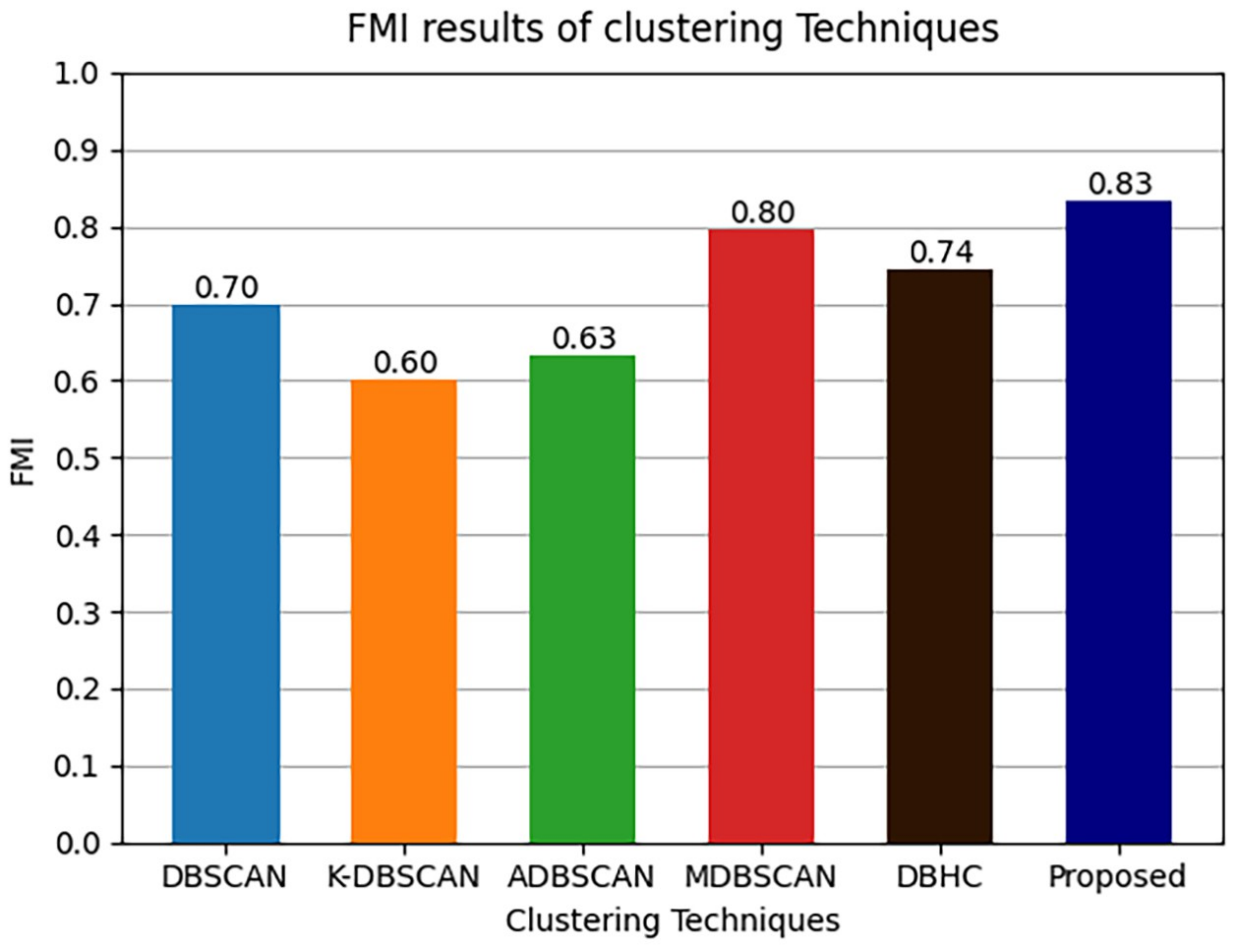
FMI metrics results.

**Fig 15:**
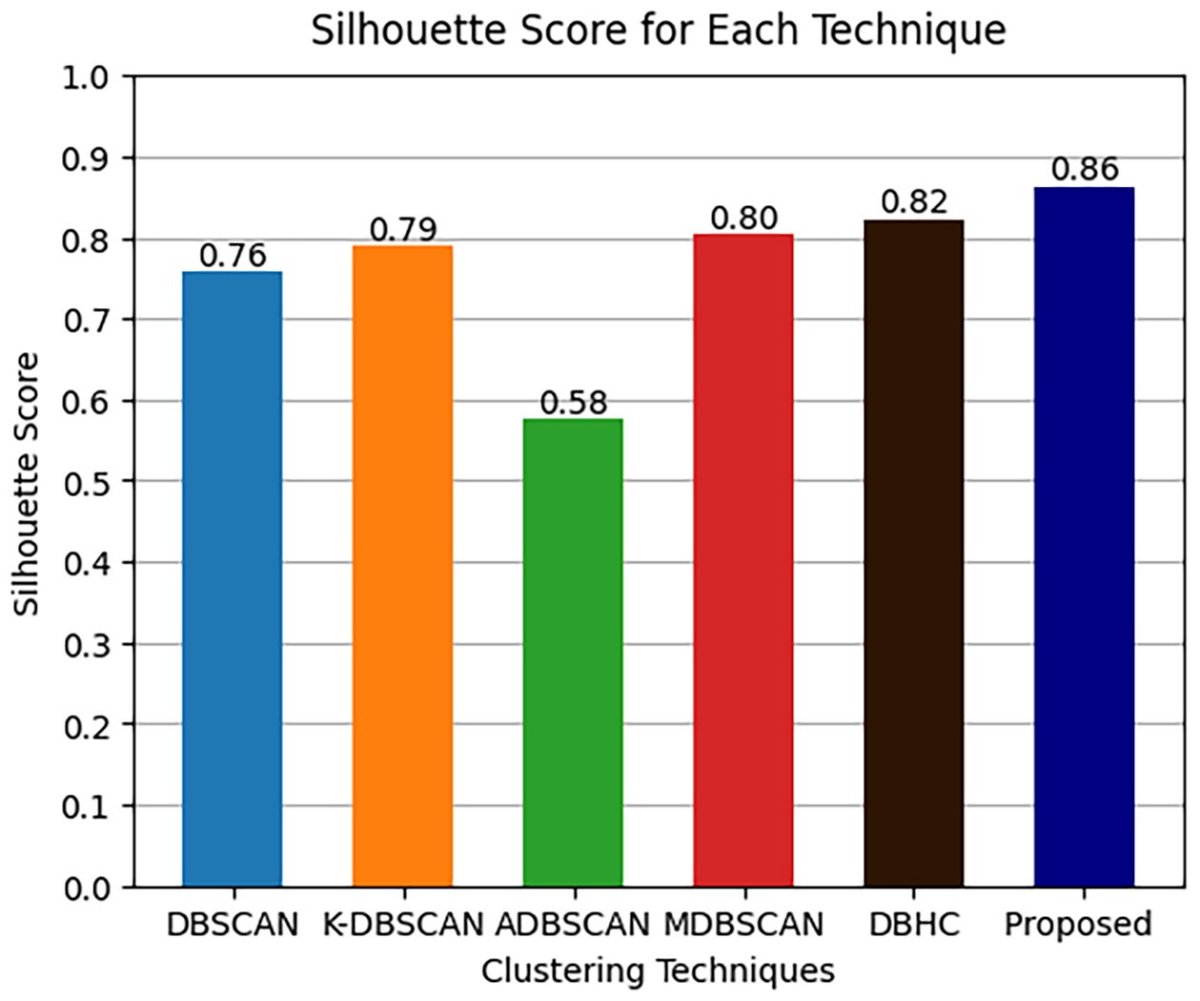
Silhouette score metrics results.

The results of clustering metrics ARI, FMI and Silhouette score of comparative techniques along with proposed technique are presented in Figs 13–15 respectively.

Where it is shown that ADBSCAN does not perform well in ARI metric among other techniques the reason of low ARI is could be because ADBSCAN misclustered and made only six clustered therefore the ARI is compromised. Unlike ADBSCAN the MDBSCAN performed better in ARI metric as presented below MDBSCAN got 0.79 and DBHC got 0.75. In contrast DBSCAN and K-DBSCAN got 0.69 and 0.63 ARI metric score respectively. Similarly, it is shown that K-DBSCAN performed the worst in terms of FMI because it clustered two large clusters which have mis-clustered the datapoints from more than one intrusion activity therefore it got 0.60 of FMI and ADBSCAN got 0.63 of FMI. Unlike ADBSCAN the MDBSCAN performed better in ARI metric as presented in Fig 13 MDBSCAN got 0.80 and DBHC got 0.74. Furthermore, DBSCAN got 0.70 and proposed technique got 0.83 ARI metric score. Moreover, the Silhouette score of ADBSCAN is the lowest, which is 0.58 performed the worst in Silhouette score because it clustered only six clusters. Similarly, ADBSCAN achieved only 0.63, conversely DBHC performed better and achieved 0.82. The highest Silhouette score is achieved by our proposed technique, which is 0.86, since the clusters are well separated and have strong cohesion between datapoints.

Details of clustering results are further presented in Table 5. The proposed technique produced the highest average silhouette score which is 0.86. Accordingly, the ARI and FMI of the model is 0.82 and 0.83. These results are significantly higher than the results produced by other techniques. These performance measures confirm that the proposed algorithm is suitable for clustering PID image dataset.

**Table 5:** Details of overall clustering results.

S#	Technique	ARI	FMI	Silhouette Score
1	DBSCAN [47]	0.69	0.70	0.76
2	K-DBSCAN [16]	0.63	0.60	0.79
3	ADBSCAN [19]	0.63	0.63	0.58
4	MDBSCAN [17]	0.79	**0.80**	**0.80**
5	DBHC [18]	0.75	0.74	**0.82**
6	Proposed Technique	**0.82**	**0.83**	**0.86**

**Fig 16:**
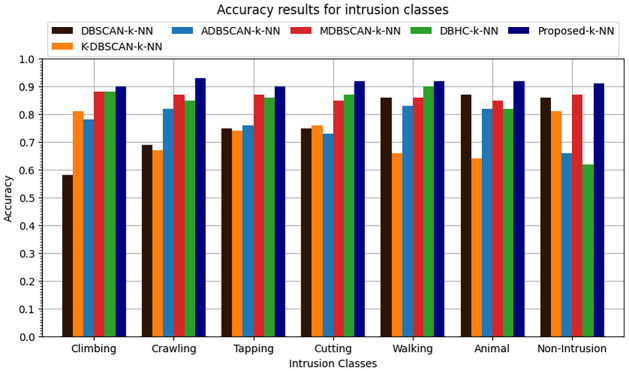
Accuracy results for seven intrusion classes.

**Fig 17:**
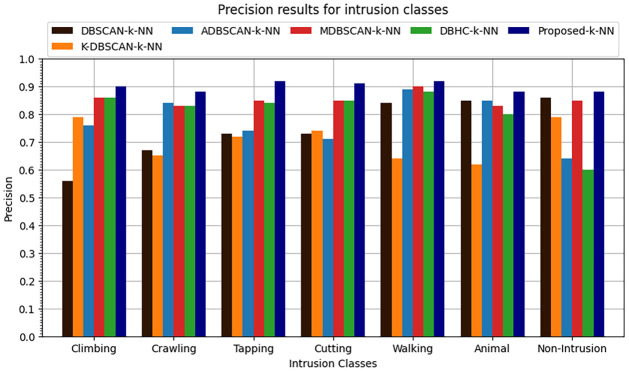
Precision results for seven intrusion classes.

**Fig 18:**
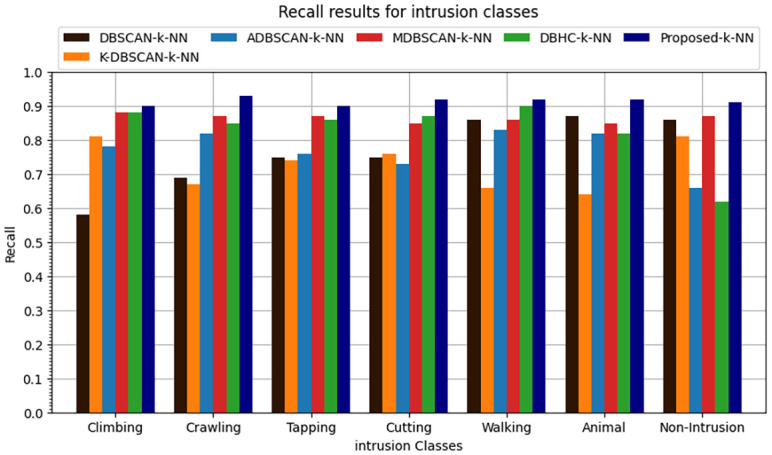
Recall results for seven intrusion classes.

**Fig 19:**
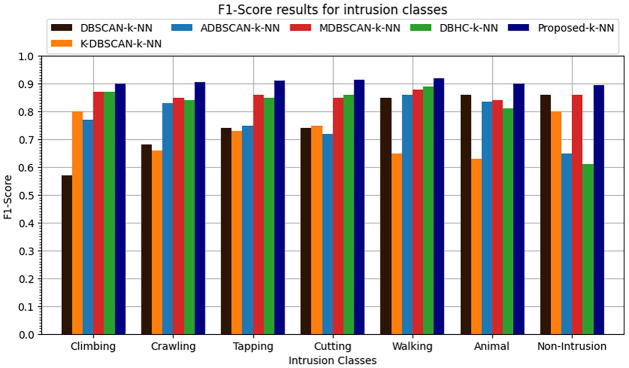
F1-score results for seven intrusion classes.

The k-NN classification algorithm is applied to the clustered data. It was found that proposed enhanced DBSCAN clustering model performs overall 0.91 accuracy, the overall precision is recorded at 0.89, recall is overall recorded as 0.91 and similarly the overall F1-score is recorded as 0.90 as shown in Figs 16–19.

## 5 Conclusion

In this research, a novel PIDS model is introduced, a groundbreaking Machine learning framework designed for perimeter intrusion detection systems. Leveraging the pre-trained Inceptionv3 model, feature extraction is performed PID image dataset, focusing on perimeter intrusion scenarios. Subsequently, employing t-SNE for dimensionality reduction and followed by density-based clustering, a substantial enhancement to the conventional DBSCAN is introduced. This enhanced version incorporates epsilon value estimation and deploys the Manhattan distance formula, optimizing point distance calculations for multi-class data. The outcomes are highly promising, with the novel PIDS model achieving an impressive silhouette score of 0.86 on the challenging PID image dataset. This success not only underscores the effectiveness of our model in accurately identifying intrusion activities but also signifies a significant advancement in PID system capabilities, paving the way for further exploration and refinement in the realm of perimeter intrusion detection. This study focuses on six types of intrusion activities, with an additional class considered as non-intrusion, resulting in a total of seven classes. Future researchers may further extend these classes. Additionally, intrusion from the top of the perimeter fence is not within the scope of this research but can be addressed in future studies.
